# B Cell–Activating Factor Promotes B Cell Survival in Ectopic Lymphoid Tissues in Nasal Polyps

**DOI:** 10.3389/fimmu.2020.625630

**Published:** 2021-01-20

**Authors:** Zhe-Zheng Wang, Jia Song, Hai Wang, Jing-Xian Li, Qiao Xiao, Ze Yu, Jin-Xin Liu, Zheng Liu

**Affiliations:** Department of Otolaryngology-Head and Neck Surgery, Tongji Hospital, Tongji Medical College, Huazhong University of Science and Technology, Wuhan, China

**Keywords:** B cell-activating factor, ectopic lymphoid tissue, nasal polyp, stromal cell, B cell

## Abstract

Ectopic lymphoid tissues (eLTs) characterized by B cell aggregation contribute to the local immunoglobulin production in nasal polyps (NPs). B cell-activating factor (BAFF) is vital for B cell survival, proliferation, and maturation. The purpose of this study is to investigate whether BAFF is involved in the B cell survival and eLT formation in NPs. The mRNA and protein levels of BAFF in NP tissues with and without eLTs were detected by PCR and ELISA assay, respectively. The cellular sources of BAFF and active caspase-3-positive B cells in NPs were studied by immunofluorescence staining. B cells purified from NP tissues were stimulated with BAFF and were analyzed by flow cytometry. Stromal cells purified from NP tissues were stimulated with lymphotoxin (LT) α_1_β_2,_ and BAFF levels in culture supernatants were analyzed by ELISA. Compared with those in control tissues and NPs without eLTs, the BAFF levels were elevated in NPs with eLTs. Abundant BAFF-positive cells and few active caspase-3-positive apoptotic B cells were found in NPs with eLTs, in contrast to those in NPs without eLTs. There was a negative correlation between the numbers of BAFF-positive cells and frequencies of apoptotic B cells in total B cells in NP tissues. BAFF protected nasal polyp B cells from apoptosis *in vitro*. Stromal cells were an important cellular source of BAFF in NPs with eLTs. LTα_1_β_2_ induced BAFF production from nasal stromal cells *in vitro*. We propose that BAFF contribute to eLT formation in NPs by promoting B cell survival.

## Introduction

Nasal polyps (NPs) are characterized by persistent inflammation of nasal and paranasal sinus mucosa with the presence of edematous outgrowth (1). More than one-third of patients with NPs are unable to achieve satisfying outcomes even with adequate medical and surgical treatments, reflecting our limited understanding of the etiology and pathogenesis of NPs and the lack of powerful therapeutic strategies (2, 3). Accumulating evidence suggest that local immunoglobulin (Ig) overproduction contributes to the local activation of mast cells, eosinophils, and complements, thus playing an important role in the pathogenesis of NPs and associating with the poor treatment outcomes (4, 5). Several studies have identified the formation of ectopic lymphoid tissues (eLTs) in NPs and revealed a critical role of eLTs in supporting local immunoglobulin production in NPs (6–10). eLT formation in NPs also correlates with poor disease control in patients with NPs (6, 8). eLTs is characterized by germinal center formation (GC) along with B cell aggregation in NPs, where B cells undergo somatic mutation, class switch recombination, and differentiation to plasma cells (6). Therefore, understanding the mechanisms underlying B cell accumulation in eLTs may be helpful to elucidate how eLTs develop in NPs.

The development of B cell aggregation depends on enhanced B cell recruitment to NP tissues and prolonged B cell survival. Our recent study found that IL-17A-induced nasal stromal cell remodeling and crosstalk between B and stromal cells *via* B cell chemokine-C-X-C motif chemokine ligand 13 (CXCL13) and lymphotoxin (LT) α_1_β_2_ have an important role in B cell recruitment and the eLT formation in NPs (9). Nevertheless, whether there is a prolonged survival of B cells in eLTs in NPs remains unknown. B cell activating factor (BAFF) is a pivotal factor for B cell survival, proliferation, and maturation (11). BAFF deficiency leads to an almost complete loss of follicular and marginal zone B lymphocytes in mice (12). On the contrary, BAFF overproduction results in B cell hyperplasia, excessive immunoglobulin production, and a glomerular nephritic syndrome or systemic lupus erythematosus–like syndrome in mice (13–15). Three cell receptors for BAFF have been identified, namely BAFF receptor (BAFF-R), transmembrane activator and cyclophilin ligand interactor, and B cell maturation antigen (16–18). Among them, the agonist effects of BAFF on B cells are mainly mediated by BAFF-R (19, 20). Previously, the increased BAFF levels have been reported in NP tissues, correlating with the numbers of B cells as well as the elevated local levels of IgA and IgE levels (21–23). However, whether BAFF is involved in the survival of B cells and formation of eLTs in NPs is still an open question.

In this study, we aim to investigate the possible role of BAFF in B cell survival and eLT formation in NPs. We explored the association between BAFF levels and B cell apoptosis in NPs, with or without eLT formation. We also determined the cellular sources of BAFF in NPs and explored the effect of BAFF on nasal polyp B cell apoptosis *in vitro*. Finally, we studied the regulation of BAFF production in nasal stromal cells.

## Materials and Methods

### Subjects and Sample Collection

This study was approved by the Ethics Committee of Tongji Hospital and conducted with written informed consent from every patient (IRB Number: TJ-C20170301). A total of 63 patients with NPs and 16 control subjects were recruited. The diagnosis of NPs was made according to the current European Position Paper on Rhinosinusitis and Nasal Polyps 2020 (1). Patients undergoing septoplasty because of anatomic variation and without other sinonasal diseases were enrolled as control subjects. Atopic status was evaluated by using skin prick test with a panel of inhalant allergens common in our region and/or by using the ImmunoCAP to detect IgE antibodies against common inhalant allergens (Phadia, Uppsala, Sweden) (24). The diagnosis of allergic rhinitis was made based on the concordance between the atopic status and typical allergic symptoms. The diagnosis of asthma was made based on history and physician’s diagnosis according to Global Initiative for Asthma guidelines (25). Intranasal steroid sprays and oral glucocorticoids were stopped at least 1 month and 3 months before surgery, respectively. None had received antileukotrienes or immunotherapy. Subjects who had cystic fibrosis, antrochoanal polyps, fungal sinusitis, gastroesophageal reflux disease, primary ciliary dyskinesia, vasculitis, or an acute upper respiratory tract infection within 1 month of entering the study were excluded from the study. None of the patients had a history of aspirin sensitivity. Symptoms including nasal obstruction, rhinorrhea, hyposmia, and facial pain/pressure were scored on a visual analog scale (VAS) of 0 to 10, with 0 indicating “no complaint whatsoever” and 10 indicating “the worst imaginable complaint” (1). The total VAS symptom scores were calculated based on the sum of these four VAS symptom domains. The overall disease burden was also scored by asking patients how troublesome their disease was in affecting daily activity and sleep on a VAS as mentioned above. Endoscopic physical findings were scored according to the Lund and Kennedy (1). Computed tomography (CT) scans were graded based on the Lund-Mackay scoring system as previously described (1).

During surgery, NP tissues were collected from patients with NPs and inferior turbinate mucosal tissues were collected from control subjects. Nasal epithelial cells were scraped from the NP tissues of patients with NPs as previously described (26, 27). Due to the limited amount of tissue samples, not all samples were included in every experimental protocol. The numbers of subjects involved in each experiment are indicated in figures or figure legends. Demographic and clinical data of subjects are summarized in **Supplementary Table S1**.

### Immunofluorescence Staining

Each nasal tissue sample was divided into at least two parts. One part was embedded in paraffin, the others were used for real-time polymerase chain reaction (PCR), enzyme-linked immunosorbent assay (ELISA), and cell isolation experiments.

Paraffin tissue sections (4 μm) were prepared from tissue blocks. One of every 10 paraffin sections was stained with hematoxylin-eosin to screen for the presence of lymphoid aggregates by two independent investigators. Based on our previous study (6, 9), if a tissue section demonstrating cell aggregates with a radial cell count of greater than 5 cells, the adjacent tissue sections were selected for immunofluorescence staining. The eLTs in NPs were identified as lymphoid aggregates with GC-like structures characterized by the presence of Ki-67^+^ proliferating B cells, CD21^+^ follicular dendritic cell networks, and peripheral node addressin-positive high endothelial venules, as previously stated (6).

For immunofluorescence staining, the tissue sections were first stained with the specific primary antibodies (**Table S2** in the **Supplementary Material**) overnight at 4°C and then incubated with fluorescence-labeled secondary antibodies (**Table S3** in the **Supplementary Material**) for 1 h. Species- and subtype-matched antibodies were used as negative controls. The numbers of positive cells in tissue sections per high power field (HPF) were counted by using Image J software (NIH Image, Bethesda, MD). Ten HPFs were randomly selected and analyzed for every tissue section.

### Quantitative RT-PCR

Total RNA was extracted from tissue or cell samples by using TRIzol reagent. Single-strand cDNA was synthesized by reverse transcription. Real-time PCR was performed with SYBR Green I and specific primers (**Table S4** in the **Supplementary Material**) as previously described (6). Glucuronidase-β (GUSB) were used as a housekeeping gene for normalization. Relative gene expression was calculated by using the 2(-Delta Delta CT) method (28).

### Nasal B Cell Purification and Culture

NP tissues were dissected and single-cell suspensions were prepared by mechanical dissociation as previously described (6). NP mononuclear cells were isolated from single-cell suspensions by means of density gradient centrifugation on Lymphoprep (AXIS-SHIELD PoC AS, Oslo, Norway). B cells were isolated from mononuclear cells by positive selection using anti-CD19 microbeads (Miltenyi Biotec, San Diego, CA, USA) according to the manufacturer’s protocol (29). The purity of the isolated cells was more than 95%, as shown in **Supplementary Figure S1A**.

B cells were cultured in RPMI 1640 (Thermo Fisher Scientific, Waltham, MA, USA) supplemented with 1% penicillin and 1% streptomycin (Thermo Fisher Scientific), and 10% FCS (GE Healthcare). B cells were stimulated with human recombinant BAFF at 10, 50, or 200 ng/ml (R&D Systems, Minneapolis, MN, USA). After 48-h stimulation, cells were harvested for flow cytometry.

### Nasal Stromal Cell Purification and Culture

Nasal stromal cells were isolated from NP single-cell suspensions by negative selection using anti-CD45 and anti-epithelial cell adhesion molecule microbeads (Miltenyi Biotec) to deplete hematopoietic and epithelial cells, as previously stated (30, 31). The purity of the isolated cells was more than 95%, as shown in **Supplementary Figure S1B**.

Isolated nasal stromal cells were cultured overnight in Dulbecco’s Modified Eagle Medium (DMEM) with 1g/L D-Glucose medium (Thermo Fisher Scientific) supplemented with 1% penicillin and 1% streptomycin (Thermo Fisher Scientific), 0.05 mg/ml gentamicin (Thermo Fisher Scientific), 10 mM 4-(2-hydroxyethyl)-1-piperazineethanesulfonic acid buffer (Thermo Fisher Scientific), 2 mM L-glutamine (Thermo Fisher Scientific), and 10% FCS (GE Healthcare). Non-adherent cells were removed on the next day. The remaining cells were cultured for 2 days and non-adherent cells were removed again. Adherent cells were grown for an additional 2 days and then treated with human recombinant LTα_1_β_2_ at 1, 10, 100, or 1000 ng/ml (R&D Systems). After 12-h stimulation, cells were harvested for real-time PCR assay. After 48-h stimulation, cell culture supernatants were collected for ELISA.

### Flow Cytometry

Cells were stained with fixable viability stain 700 (BD Bioscience, San Jose, CA, USA) to exclude dead cells. For cell surface staining, FITC-labeled anti-CD19 antibody (1:100; clone: HIB19; BD Bioscience) were incubated with cells for 30 min at 4°C. For intracellular active caspase-3 staining, cells were permeabilized by using Cytofix/Cytoperm (BD Biosciences) for 20 min on ice after surface staining. Then cells were stained with PE-labeled anti-active caspase-3 antibody (1:100; clone: C92-605.1; BD Biosciences) for 30 min at 4°C. Cells were enumerated using a BD LSR Fortessa™ X-20 (BD Bioscience) instrument and data were analyzed using FlowJo software (TreeStar, Ashland, OR, USA).

### Enzyme-Linked Immunosorbent Assay

The BAFF protein levels in tissue homogenates and cell culture supernatants were measured by using a commercial enzyme-linked immunosorbent assay (ELISA) kit (Boster Biotechnology, Wuhan, China), and the lower detection limit was 2 pg/ml. The tissue homogenates were generated by mechanical dissociation as previously mentioned (6). The levels of BAFF in tissues were normalized to total tissue protein levels quantified by using a BCA protein detection kit (Guge Biotechnology, Wuhan, China).

### Statistical Analysis

Statistical analysis was performed with the SPSS 18.0 software. Data distribution was evaluated by the Shapiro Wilk test to assess the normality and homogeneity of variance. For normally distributed data, ANOVA with a Tukey’s *post-hoc* test for multiple comparisons and Student’s *t* test for binary comparisons were applied. For non-normally distributed data, a Kruskal-Wallis test with a Dunn’s *post-hoc* test for multiple comparisons and Mann-Whitney U test for binary comparisons were employed. The Spearman rank test was used for correlations. Chi-square test or Fisher’s exact test was applied to analyze differences in proportions between groups. Data derived from tissue studies are presented in dot plots unless specifically stated. Symbols represent individual samples, horizontal bars represent medians, and error bars show interquartile ranges. Data from cell culture experiments are expressed as means ± SEMs. Significance was accepted at a *P* value of less than 0.05.

## Results

### Increased BAFF Levels in NPs With eLTs

**Figure 1:**
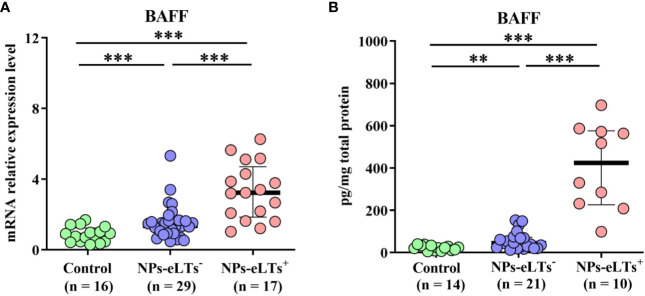
The elevated expression levels of BAFF in NPs with eLTs. **(A)** The mRNA expression levels of BAFF in control tissues, NPs without eLTs, and NPs with eLTs as detected by real-time PCR assay. **(B)** The protein levels of BAFF in control tissues, NPs without eLTs, and NPs with eLTs as detected by ELISA. BAFF, B cell-activating factor; NPs, nasal polyps; eLTs^+^, with ectopic lymphoid tissues; eLTs^-^, without ectopic lymphoid tissues. **P < 0.01; ***P < 0.001.

Consistent with previous studies (21–23), we found that although compared to those in control tissues, BAFF levels were elevated in both NP tissues with and without eLTs at the mRNA and protein level detected by real-time PCR and ELISA assays, respectively, there was a further increase of BAFF expression in NP with eLTs in comparison to that in NP without eLTs (**Figures 1A, B**).

### Decreased Apoptosis of B Cells Associates With Elevated BAFF Levels in NPs With eLTs

**Figure 2:**
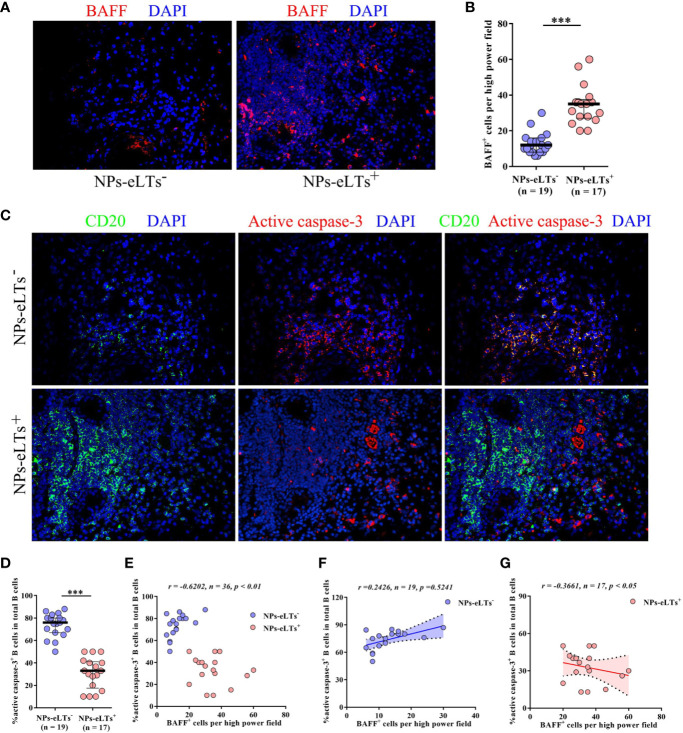
Elevated BAFF expression associates with decreased apoptosis of B cells in NPs with eLTs. **(A)** Immunofluorescence staining of NP tissues shows BAFF-positive cells in NPs with and without eLTs. Original magnification × 400. **(B)** The numbers of BAFF^+^ cells per high power filed in NPs with or without eLTs. **(C)** Double immunofluorescence staining of consecutive NP tissue sections of those shown in **Figure 2A** demonstrates active caspase-3^+^CD20^+^ B cells in NPs with and without eLTs. Original magnification × 400. **(D)** The percentages of active caspase-3^+^CD20^+^ B cells in total CD20^+^ B cells in NPs with or without eLTs. **(E–G)** The correlation between the percentages of active caspase-3^+^CD20^+^ B cells in total B cells and the numbers of BAFF^+^ cells in total NPs **(E)**, NPs without eLTs **(F)**, and NPs with eLTs **(G)**. BAFF, B cell-activating factor; NPs, nasal polyps; eLTs^+^, with ectopic lymphoid tissues; eLTs^-^, without ectopic lymphoid tissues. ***P < 0.001.

BAFF has a fundamental role in B cell survival (11, 32, 33). To explore whether increased BAFF has a role in B cell survival in NPs with eLTs, we studied the relationship between BAFF^+^ cells and active caspase-3^+^CD20^+^ apoptotic B cells in NP tissues. In line with real-time PCR and ELISA findings, we found that numbers of BAFF^+^ cells in cell aggregates in lamina propria were increased in NPs with eLTs compared to those in NPs without eLTs (**Figures 2A, B**). In addition, we found that although NPs with eLTs had significantly more B cells than NPs without eLTs, fewer active caspase-3^+^CD20^+^ apoptotic B cells were found in NPs with eLTs (**Figure 2C**). The frequencies of active caspase-3^+^CD20^+^ apoptotic B cells in total CD20^+^ B cells in NPs with eLTs were significantly lower than those in NPs without eLTs (**Figure 2D**). Moreover, there was an inverse correlation between the numbers of BAFF^+^ cells and the percentages of apoptotic B cells in total NPs (**Figure 2E**). We also analyzed the relationship between BAFF expression and B cell apoptosis in different types of NPs separately. In NPs without eLTs, possibly due to the low levels of BAFF, we did not observe significant correlation between the numbers of BAFF^+^ cells and the percentages of apoptotic B cells (**Figure 2F**). In NPs with eLTs, we did find an inverse correlation between the numbers of BAFF^+^ cells and percentages of apoptotic B cells (**Figure 2G**).

### BAFF Rescues NP B Cells From Apoptosis

**Figure 3:**
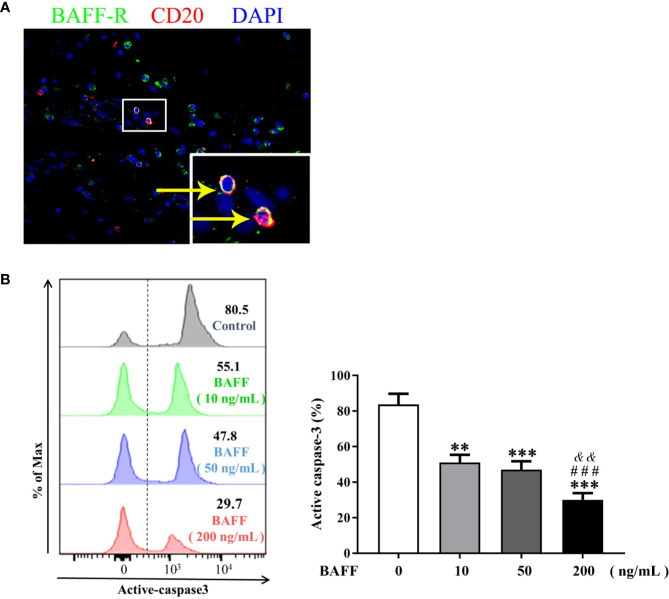
BAFF treatment rescues nasal polyps (NP) B cells from apoptosis. **(A)** Double immunofluorescence staining of NP tissues shows BAFF-R expression on CD20^+^ B cells. Original magnification × 400. Inset shows a higher magnification of the outlined area, and arrows denote positive cells. **(B)** B cells isolated from NP tissues were treated with 10, 50, and 200 ng/ml BAFF for 48 h. The frequencies of active caspase-3^+^ B cells in CD19^+^ B cells were detected by flow cytometry (n = 8). The representative flow plots are shown. BAFF, B cell-activating factor; BAFF-R, B cell-activating factor receptor. ** indicates *P* < 0.01 vs. control (without BAFF treatment); *** indicates *P* < 0.001 vs. control (without BAFF treatment); ^###^ indicates *P* < 0.001 vs. 10 ng/ml BAFF-treated group; ^&&^ indicates *P* < 0.01 vs. 50 ng/ml BAFF-treated group.

To further elucidate the role of BAFF in the survival of NP B cells, we first confirmed the expression of BAFF-R on B cells in NP tissues by immunoflurescence staining (**Figure 3A**). After 48-h *in vitro* culture, majority of NP B cells (mean, 82.5%) underwent spontaneous apoptosis (**Figure 3B**). Of interest, BAFF dose-dependently suppressed the apoptosis of NP B cells, as indicated by the decreased frequencies of active caspase-3^+^CD19^+^ apoptotic B cells *in vitro* with BAFF treatment compared to those without BAFF treatment (**Figure 3B**).

### Stromal Cells Are an Important Cellular Source of BAFF Expression in NPs With eLTs

**Figure 4:**
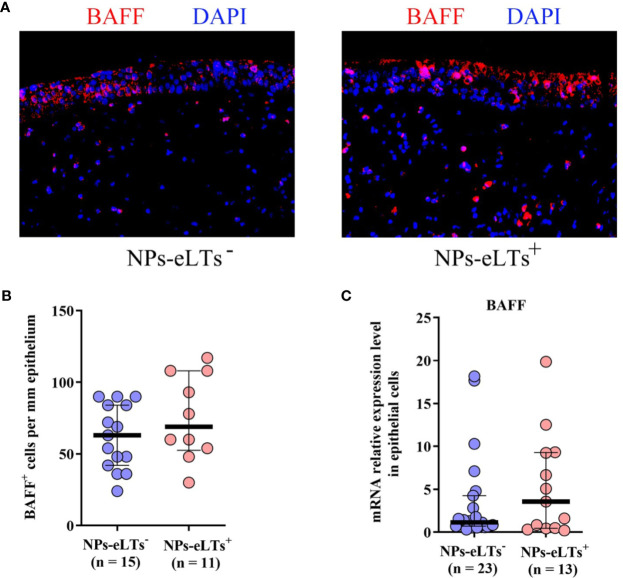
The expression of BAFF in nasal epithelial cells in NPs with or without eLTs. **(A)** Representative photomicrographs shows that BAFF immunoreactivity in both nasal epithelial cells and infiltrating cells in lamina propria in NPs with and without eLts. Original magnification × 400. **(B)** The numbers of BAFF^+^ cells in the epithelium in NPs with or without eLTs. **(C)** The BAFF mRNA expression in nasal epithelial cells in NPs with and without eLTs. BAFF, B cell-activating factor; NPs, nasal polyps; eLTs^+^, with ectopic lymphoid tissues; eLTs^-^, without ectopic lymphoid tissues.

We then explored the cellular sources of BAFF in NPs with eLTs. Previous studies have reported BAFF expression in nasal epithelial cells and infiltrating cells in lamina propria in NPs (21–23). By immunofluorescence staining, we also found BAFF immunoreactivity in both epithelial cells and infiltrating cells in lamina propria in NPs (**Figure 4A**). Nevertheless, we did not find a significant difference in the numbers of BAFF^+^ cells (**Figure 4B**) and BAFF mRNA levels (**Figure 4C**) in nasal epithelial cells between NPs with and without eLTs.

**Figure 5:**
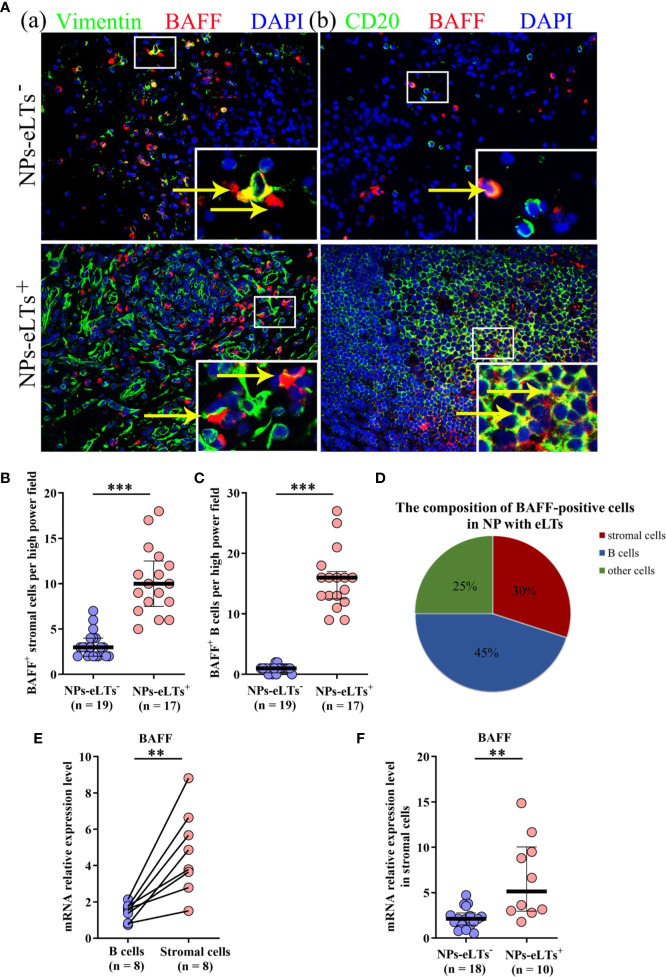
The expression of BAFF in lamina propria in NPs with or without eLTs. **(A)** Double immunofluorescence staining of NP tissues showing BAFF expression on vimentin^+^ stromal cells (a) and CD20^+^ B cells (b) in NPs with and without eLTs. Original magnification × 400. Insets show a higher magnification of the outlined area, and arrows denote positive cells. **(B, C)** The numbers of BAFF^+^ stromal cells **(B)** and BAFF^+^ B cells **(C)** in NPs with or without eLTs. **(D)** The cell type composition of BAFF^+^ cells in NPs with eLTs. **(E)** The relative mRNA expression of BAFF in stromal cells and B cells isolated from same samples of NP tissues with eLTs. **(F)** The BAFF mRNA expression in stromal cells purified from NPs with and without eLTs. BAFF, B cell-activating factor; NPs, nasal polyps; eLTs^+^, with ectopic lymphoid tissues; eLTs^-^, without ectopic lymphoid tissues. **P < 0.01; ***P < 0.001.

In addition to epithelial cells, various immune cells have been found expressing BAFF, including mast cells, neutrophils, stromal cells, B cells, and T cells (34–36). By double immunofluorescence staining, we found that BAFF was mainly expressed by stromal cells and B cells, and there were a few of BAFF^+^ neutrophils, mast cells and T cells in NPs, especially in NPs with eLTs (**Figure 5A**; **Supplementary Figure S2**). The numbers of BAFF^+^ stromal cells and B cells were elevated in NPs with eLTs compared to those in NPs without eLTs (**Figures 5B, C**). In NPs with eLTs, 30% (mean) BAFF^+^ cells were stromal cells and 45% (mean) of them were B cells (**Figure 5D**).

Since BAFF-R is expressed by B cells, the BAFF expression detected by immunofluorescence staining might be interfered by the BAFF binding to the BAFF-R on B cells. We therefore purified B cells and stromal cells from NPs with eLTs and detected the mRNA production of BAFF in these two types of cells. We discovered that stromal cells had more abundant BAFF mRNA production than B cells in NPs with eLTs (**Figure 5E**), suggesting that stromal cells have a higher capacity than B cells to produce BAFF in NPs with eLTs. Furthermore, when comparing the difference between stromal cells in NPs with and without eLTs, we found that the BAFF mRNA expression was elevated in stromal cells isolated from NPs with eLTs in comparison to those isolated from NPs without eLTs (**Figure 5F**).

### LTα_1_β_2_ Induces the BAFF Production From Nasal Stromal Cells

**Figure 6:**
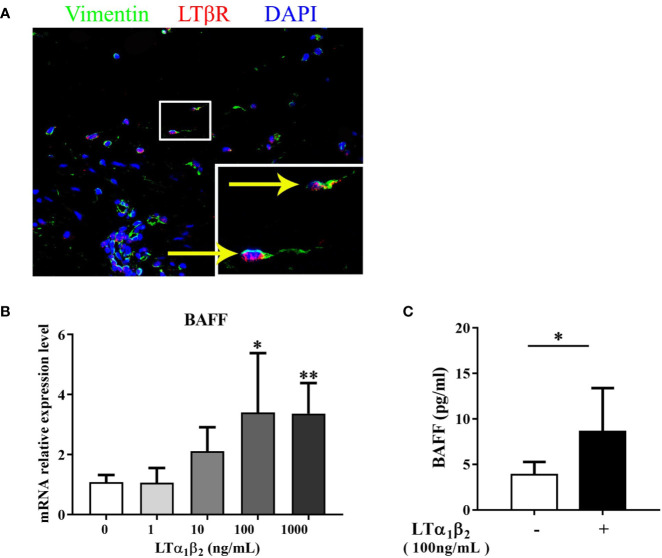
LTα_1_β_2_ induces the production of BAFF from nasal stromal cells. **(A)** Immunofluorescence staining of nasal polyps (NP) tissues showing LTβR expression on vimentin^+^ stromal cells. Original magnification ×400. Inset shows a higher magnification of the outlined area, and arrows denote positive cells. **(B)** Stromal cells purified from NP tissues were treated with LTα_1_β_2_ at various doses for 12 h, and the mRNA expression of BAFF in stromal cells was detected by real-time PCR (n = 7). **(C)** The protein levels of BAFF in stromal cell culture supernatants after LTα_1_β_2_ (100 ng/ml) treatment for 48 h (n = 7). BAFF, B cell-activating factor; LTβR, lymphotoxin β receptor; LTα_1_β_2_, lymphotoxin α_1_β_2_. * indicates *P* < 0.05 vs. control (without LTα1β_2_ treatment); ** indicates *P* < 0.01 vs. control (without LTα_1_β_2_ treatment).

Our recent study demonstrated that LTα_1_β_2_-LTβ receptor (LTβR) pathway induced CXCL13 production by nasal stromal cells and initiated the lymphocyte recruitment during the eLT development in NPs (9). The increased expression of LTα_1_β_2_ in NPs with eLTs has been evidenced in our previous studies (6, 9). Here, we explored whether LTα_1_β_2_ contributes to the increased production of BAFF in stromal cells in NPs with eLTs. LTβR expression was again confirmed in nasal stromal cells in NPs by immunofluorescence staining (**Figure 6A**). LTα_1_β_2_ induced BAFF mRNA expression in stromal cells isolated from NP tissues in a dose-dependent manner *in vitro* (**Figure 6B**). Consistently, the enhanced BAFF protein levels in culture supernatants of nasal stromal cells were found after stimulation with LTα_1_β_2_ at 100 ng/ml (**Figure 6C**).

For immunofluorescence staining experiemtnts, the representative images showing the staining for the isotype control antibodies are presneted in **Supplementary Figure S3**.

### The Associations Between BAFF Expression and Clinical Features

Our previous study has demonstrated the associations between eLT formation and disease duration, symptom severity and prior surgery history in patients with NPs (6). In this study, we further found that the protein levels of BAFF positively correlated with disease duration and overall disease burden in patients with NPs (**Table 1**). In addition, we found NP patients with prior surgery history [n = 10; median and interquartile range, 177 pg/mg (96-353 pg/mg)] had higher BAFF levels than those without prior surgery (n = 21; median and interquartile range, 131 pg/mg (52-215 pg/mg)] (*P = 0.028*).

**Table 1:** The associations between the BAFF protein levels and clinical characteristics in NP patients (n = 31).

	*Correlation coefficient*	*P value*
Disease duration	***0.440***	***0.020***
Symptom VAS scores		
Nasal obstruction	*0.281*	0.203
Rhinorrhea	*0.109*	*0.581*
Facial pain	*0.039*	*0.835*
Loss of smell	*−0.017*	*0.928*
Total score	*0.272*	*0.138*
Overall burden VAS scores	***0.366***	***0.043***
Endoscopic scores	*0.146*	*0.442*
CT scores	*0.144*	*0.447*

BAFF, B cell-activating factor; NP, nasal polyps; VAS, visual analog scale; CT, computed tomography. The values in bold have a corresponding p value that is <0.05.

## Discussion

The accumulation of B cells is an important step for the development of eLTs. Although local B cell residency recruited by the elevated expression of B cell chemokines has an important role in B cell aggregation in eLTs in NPs (8), whether there is an involvement of prolonged B cell survival in eLT formation in NPs remains unclear. In this study, for the first time, we demonstrated that the apoptosis of B cells in eLTs was significantly inhibited compared to the diffuse infiltrated B cells in NPs without eLTs. The high levels of BAFF may rescue spontaneous B cell apoptosis in NPs with eLTs. Nasal stromal cells were an important cellular source of BAFF in NPs with eLTs and LTα_1_β_2_ induced BAFF production from stromal cells. Our study highlights a critical role of BAFF in the eLT formation in NPs by facilizing B cell survival.

Previous studies have noted the increased production of BAFF in NPs and the association between BAFF levels and B cell infiltration as well as local IgE and IgA levels in NPs (21–23). However, those studies did not dissect the underlying mechanisms. It has been evidenced that the local immunoglobulin levels were significantly higher in NPs with eLTs than NP without eLTs, underscoring a critical role of eLTs in supporting local immunoglobulin production in NPs (6). Therefore, it is interesting to know whether BAFF contributes to eLT formation in NPs and thus promotes the local immunoglobulin production in NPs. In line with previous studies (21–23), we here found an upregulation of BAFF production in NP tissues in comparison to control tissues. Nevertheless, of importance, we discovered a significant further increase of BAFF production in NPs with eLTs in comparison to that in NPs without eLTs, suggesting an association between BAFF and eLT formation in NPs. BAFF has previously been found involved in eLT development and disease progression in patients with chronic obstructive pulmonary diseases and lupus nephritis by promoting the survival of B cells or positioning of T cells (37–40). Previous studies have discovered that human mature B cells are devoid of apoptosis by BAFF-BAFF-R interaction on B cell antigen receptor signaling (32). We found that along with the increased numbers of BAFF-positive cells, the frequencies of apoptotic B cells were significantly reduced in NPs with eLTs. We also found that there was a strong negative correlation between the numbers of BAFF-positive cells and the frequencies of apoptotic B cells in NPs. In addition, *in vitro* study showed that BAFF was able to promote nasal B cell survival. Collectively, these several lines of evidence suggest that excessive BAFF production protects nasal B cell from apoptosis, and therefore leads to B cell aggregation and eLT formation in NPs.

BAFF can be produced by various cell types including epithelial cells, stromal cells, T cells, B cells neutrophils, and mast cells, in response to different stimulus (34, 36, 38). Stimulus such as Toll-like receptor ligands, microbes, allergens, cigarette smoking, and chemicals trigger BAFF production. Proinflammatory cytokines, and Th1- and Th17-related cytokines are also able to induce BAFF production. The cellular sources of BAFF are highly tissue- and disease-dependent (21–23). In this study, although we discovered immunoreactivity of BAFF in nasal epithelial cells, we did not find a significant difference in BAFF expression in nasal epithelial cells between NPs with and without eLTs, disfavoring a role of epithelial cell-derived BAFF in eLT formation in NPs. Furthermore, we found BAFF immunoreactivity in B cells and stromal cells in NPs with eLTs. However, BAFF expression in lymphocytes detected by immunofluorescence staining should be considered with caution, since B cells have BAFF-R expression and the cell membrane-bound BAFF may interfere the detection. Indeed, despite that B cells accounted for higher percentages of BAFF^+^ cells than stromal cells (45% vs. 30%) in NPs with eLTs, isolated nasal stromal cells had more abundant production of BAFF mRNA than B cells, suggesting that nasal stromal cells may have a higher capacity to produce BAFF than B cells in NPs. In addition, the production of BAFF was elevated in stromal cells in NPs with eLTs than those in NPs without eLTs, suggesting that nasal stromal cells are an important cellular source of BAFF in NPs with eLTs. Stromal cells provide a structural and functional niche for lymphocyte migration, survival, antigen encounter, and tolerance in lymphoid tissues (41, 42). Our recent study found that stromal cells may initiate B cell recruitment by secreting CXCL13, thus play an important role in the early stage of eLT development (9). In the development of SLOs, LTα_1_β_2_-LTβR pathway is crucial for stromal cell expansion, differentiation, and maturation (43–45). Here, we found that LTα_1_β_2_ induced the BAFF production from nasal stromal cells. Therefore, stromal cells not only contribute to the B cell recruitment in the initial stage of eLT formation, but also prolong B cell survival and further promote eLT expansion in NPs.

Consisitent with our previous findings regarding the relationship between eLT formation and clinical features (6), we found that BAFF expression was also correlated with disease duration, overall disease burden and prior surgery in patients with NPs, further highlighting a role of BAFF in the disease progression in patients with NPs.

NPs may have different phenotypes/endotypes. NPs can be classified into eosinophilic and noneosinophilic types based on the extent of eosinophilic inflammation, and these two types of NPs demonstrate several differences in immunological and inflammatory features (46, 47). Our previous study found that the frequencies of eLT formation were similar in eosinophilic and noneosinophilic NPs, and the expression of homeostatic chemokines and lymphorganogenic molecules, including BAFF, CXCL13, and LT, was shared by eosinophilic and noneosinophilic NPs with eLTs, suggesting a similar molecular pathway for lymphoid neogenesis in both types of NPs (6, 9). Therefore, similar to our recent published study (9), we did not further subclassify NPs into eosinophilic and noneosinophilic types in this study

In summary, our study suggests that overproduction of BAFF from nasal stromal cells induced by LTα_1_β_2_ may rescue B cell from apoptosis and therefore contribute to B cell aggregation and eLT formation in NPs. Targeting BAFF may have therapeutic potential in preventing or alleviating the development of eLTs and chronic inflammation in patients with NPs. The BAFF inhibitor, belimumab, has been approved for the treatment of system lupus erythematosus patients characterized by IgG overproduction and eLT formation, and exhibits modest, but durable, efficacy in decreasing disease flares and organ damage (48, 49). It will be of great interest to test the efficacy of belimumab in the treatment of refractory NPs or NPs with eLTs in future.

## Data Availability Statement

The original contributions presented in the study are included in the article/**Supplementary Material**. Further inquiries can be directed to the corresponding author.

## Ethics Statement

The studies involving human participants were reviewed and approved by IRB Number: TJ-C20170301. The patients/participants provided their written informed consent to participate in this study.

## Author Contributions

Z-ZW performed cell culture, histology, flow cytometry, ELISA and PCR assays, analyzed data, and prepared the manuscript. JS and HW performed ELISA and PCR assays. J-XLi, QX, and ZY participated in tissue sample collection. J-XLiu participated in data discussion. ZL designed the study and prepared the manuscript. All authors contributed to the article and approved the submitted version.

## Funding

This study was supported by the National Natural Science Foundation of China (NSFC) grants 81630024 and 81920108011 (ZL), and 81900925 (JS).

## Conflict of Interest

The authors declare that the research was conducted in the absence of any commercial or financial relationships that could be construed as a potential conflict of interest.
