# Supplementary material for: B Cell–Activating Factor Promotes B Cell Survival in Ectopic Lymphoid Tissues in Nasal Polyps
Source: Front Immunol. 2021 Jan 20;11:625630. doi: 10.3389/fimmu.2020.625630 (PMC7854540; doi:10.3389/fimmu.2020.625630)
Supplement: Supplementary file 1 [file DataSheet_1.docx]

Supplementary Material

**B cell–activating factor promotes B cell survival in ectopic lymphoid tissues in nasal polyps**

Zhe-Zheng Wang, M.D.^1^, Jia Song, M.D., Ph.D.^1^, Hai Wang, M.D., Ph.D.^1^, Jing-Xian Li, M.D.^1^, Qiao Xiao, M.D.^1^, Ze Yu, M.D.^1^, Jin-Xin Liu, M.D., Ph.D.^1^ Zheng Liu, M.D., Ph.D.^1*^

^1^Department of Otolaryngology-Head and Neck Surgery, Tongji Hospital, Tongji Medical College, Huazhong University of Science and Technology, Wuhan, China

***For correspondence, please contact:**

Zheng Liu, M.D., Ph.D.
Department of Otolaryngology-Head and Neck Surgery
Tongji Hospital, Tongji Medical College
Huazhong University of Science and Technology
No. 1095 Jiefang Avenue
Wuhan 430030, China

Tel: 86-27-83663529; Fax: 86-27-83663529
E-mail: [zhengliuent@hotmail.com](mailto:zhengliuent@hotmail.com)

**Supplementary Figures and Tables**

**Table S1. Patients’ demographic characteristics**

|  | **Control** | **NPs without eLTs** | **NPs with eLTs** | ***P value*** | |
| --- | --- | --- | --- | --- | --- |
| **Total subject number** | 16 | 40 | 23 |  |  |
| **Methodology used** |  |  |  |  |  |
| ***Immunofluorescence staining*** |  |  |  |  |  |
| Subject number, N |  | 19 | 17 |  |  |
| Gender, male, N (%) |  | 11 (58%) | 11 (64%) | *0.676* |  |
| Age, years, median (IQR) |  | 38 (30, 51) | 46 (30, 58) | *0.723* |  |
| Patients with atopy, N (%) |  | 3 (16%) | 3 (18%) | *1* |  |
| Patients with AR, N (%) |  | 2 (11%) | 2 (12%) | *1* |  |
| Patients with asthma, N (%) |  | 1(5%) | 1 (6%) | *1* |  |
| ***Real-time PCR*** |  |  |  |  |  |
| Subject number, N | 16 | 29 | 17 |  |  |
| Gender, male, N (%) | 10 (63%) | 16 (55%) | 11 (64%) | *0.797* |  |
| Age, years, median (IQR) | 33 (21, 49) | 41 (30, 64) | 46 (30, 58) | *0.745* |  |
| Patients with atopy, N (%) | 1 (6%) | 5 (17%) | 3 (18%) | *0.651* |  |
| Patients with AR, N (%) | 0 (0) | 3 (10%) | 2 (12%) | *0.582* |  |
| Patients with asthma, N (%) | 0 (0) | 1 (3%) | 1 (6%) | *1* |  |
| ***ELISA*** |  |  |  |  |  |
| Subject number, N | 14 | 21 | 10 |  |  |
| Gender, male, N (%) | 8 (57%) | 13 (61%) | 7 (70%) | *0.922* |  |
| Age, years, median (IQR) | 38 (26, 48) | 43 (30, 55) | 46 (35, 56) | *0.852* |  |
| Patients with atopy, N (%) | 2 (14%) | 5(24%) | 2 (20%) | *0.889* |  |
| Patients with AR, N (%) | 0 (0) | 3 (14%) | 1 (10%) | *0.421* |  |
| Patients with asthma, N (%) | 0 (0) | 1 (5%) | 1 (10%) | *0.703* |  |
| ***Cell culture study*** |  |  |  |  |  |
| Subject number, N |  | 10 | 5 |  |  |
| Gender, male, N (%) |  | 8 (60%) | 3 (60%) | *0.560* |  |
| Age, years, median (IQR) |  | 43 (32, 60) | 48 (42, 53) | *0.957* |  |
| Patients with atopy, N (%) |  | 2 (20%) | 1 (20%) | *1* |  |
| Patients with AR, N (%) |  | 1 (10%) | 1 (20%) | *1* |  |
| Patients with asthma, N (%) |  | 1 (10%) | 1 (20%) | *1* |  |

NPs, nasal polyps; AR, allergic rhinitis; eLTs, ectopic lymphoid tissues; IQR, interquartile range.

**Table S2. Primary antibodies used in immunofluorescence staining**

| **Antibody** | **Species** | **Concentration** | **Clone** | **Reference** | **Source** |
| --- | --- | --- | --- | --- | --- |
| CD20 | Mouse | Undiluted | L26 | ZM-0039 | Zhongshan Golden Bridge Biotechnology (Beijing, China) |
| CD20 | Rabbit | Undiluted | EP7 | ZA-0549 | Zhongshan Golden Bridge Biotechnology |
| CD3 | Rabbit | Undiluted | EP41 | ZA-0503 | Zhongshan Golden Bridge Biotechnology |
| LTβR | Rabbit | 1:100 | polyclonal | GTX12264 | GeneTex (Irvine, Southern California, USA) |
| Vimentin | Mouse | 1:100 | V9 | ab8069 | Abcam (Cambridge, MA, UK) |
| Vimentin | Rabbit | 1:100 | polyclonal | ab45939 | Abcam |
| MPO | Rabbit | 1:100 | EPR20257 | ab208670 | Abcam |
| Tryptase | Rabbit | 1:100 | EPR9522 | ab151757 | Abcam |
| Active caspase 3 | Rabbit | 1:100 | polyclonal | ab2302 | Abcam |
| BAFF | Rat | 1:100 | Buffy 2 | ab16081 | Abcam |
| BAFF-R | Rabbit | 1:200 | polyclonal | ab5965 | Abcam |

BAFF, B cell-activating factor; BAFF-R, B cell-activating factor receptor; LTβR, lymphotoxin β receptor, MPO, Myeloperoxidase.

**Table S3. Secondary antibodies used in immunofluorescence staining**

| **Antibody** | **Concentration** | **Clone** | **Reference** | **Source** |
| --- | --- | --- | --- | --- |
| IFKine™ Red donkey anti-mouse IgG | 1:100 | polyclonal | A24411-1 | Abbkine (Wuhan, China) |
| IFKine™ Green donkey anti-mouse IgG | 1:100 | polyclonal | A24211-1 | Abbkine |
| IFKine™ Red donkey anti-rabbit IgG | 1:100 | polyclonal | A24421-1 | Abbkine |
| IFKine™ Green donkey anti-rabbit IgG | 1:100 | polyclonal | A24221-1 | Abbkine |
| IFKine™ Red goat anti-rat IgG | 1:100 | polyclonal | A23440-1 | Abbkine |
| IFKine™ Green goat anti-rat IgG | 1:100 | polyclonal | A23240-1 | Abbkine |

**Table S4. Primers used in RT-PCR analysis**

| **Primer** | **Primer sequences** | **Annealing temperature (°C)** | **Expected product**  **size (bp)** |
| --- | --- | --- | --- |
| BAFF | (F) 5’- CCTCACGGTGGTGTCTTTCTA -3’ | 60 | 226 |
|  | (R) 5’- AACGGCACGCTTATTTCTGCT -3’ |  |  |
| GUSB | (F) 5’-GACACGCTAGAGCATGAGGG-3’ | 60 | 121 |
|  | (R) 5’-GGGTGAGTGTGTTGTTGATGG-3’ |  |  |

BAFF, B cell-activating factor; GUSB, glucuronidase-β.

**FIGURE LEGENDS**

**Supplementary Fig S1. Representative photomicrographs show the purity of isolation of B cells (A) and stromal cells (B) from NP tissues.** EpCAM, epithelial cell adhesion molecule.

**Supplementary Fig S2. The expression of BAFF in MPO^+^ neutrophils (a), CD3^+^ T cells (b), and tryptase^+^ mast cells (c) in NPs with or without eLTs.** Representative Immunoﬂuorescence staining of tissue sections are shown. Original magniﬁcation × 400. BAFF, B cell-activating factor; NPs, nasal polyps; eLTs^+^, with ectopic lymphoid tissues; eLTs^-^, without ectopic lymphoid tissues; MPO, myeloperoxidase.

**Supplementary Fig S3. Representative photomicrograph showing the staining for the isotype control antibodies.** Original magniﬁcation × 400. BAFF, B cell-activating factor; BAFF-R, B cell-activating factor receptor; LTβR, lymphotoxin β receptor; MPO, myeloperoxidase.
