# Supplementary figures and images for: B Cell–Activating Factor Promotes B Cell Survival in Ectopic Lymphoid Tissues in Nasal Polyps
Source: Front Immunol. 2021 Jan 20;11:625630. doi: 10.3389/fimmu.2020.625630 (PMC7854540; doi:10.3389/fimmu.2020.625630)

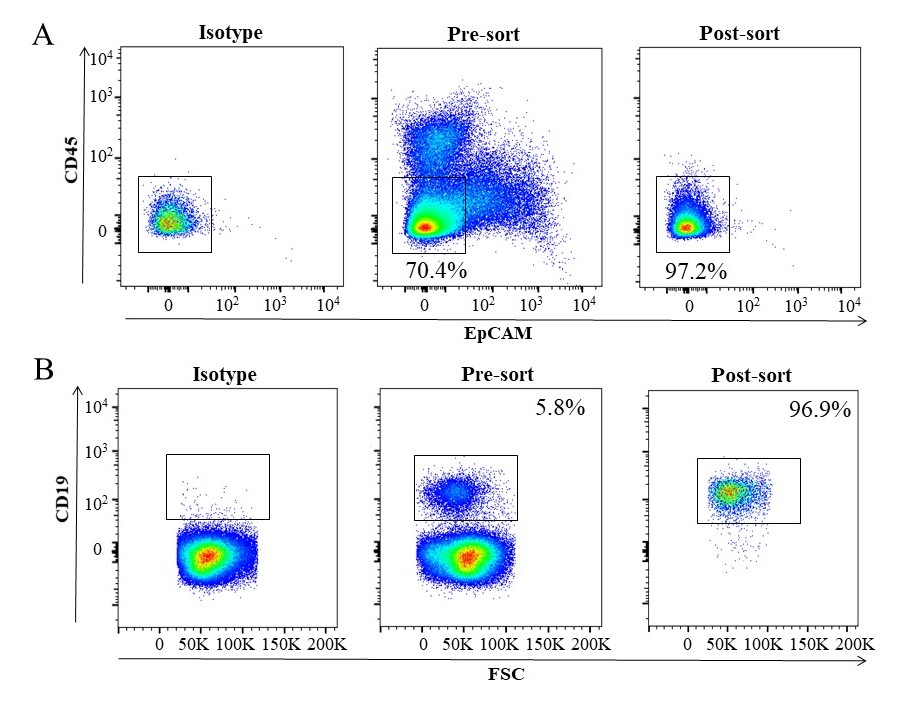

Supplement: Supplementary file 2 [file Image_1.jpeg]

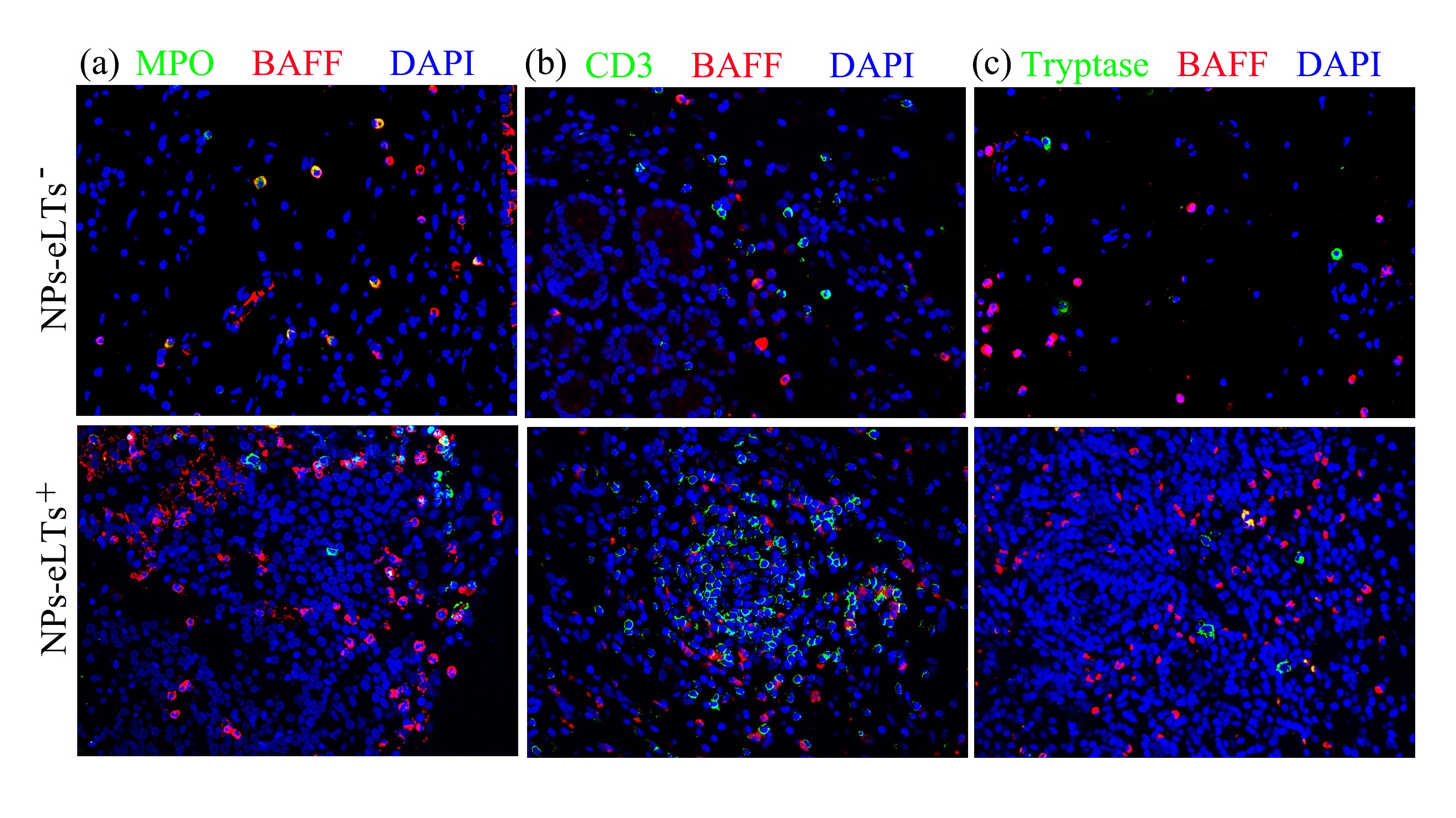

Supplement: Supplementary file 3 [file Image_2.jpeg]

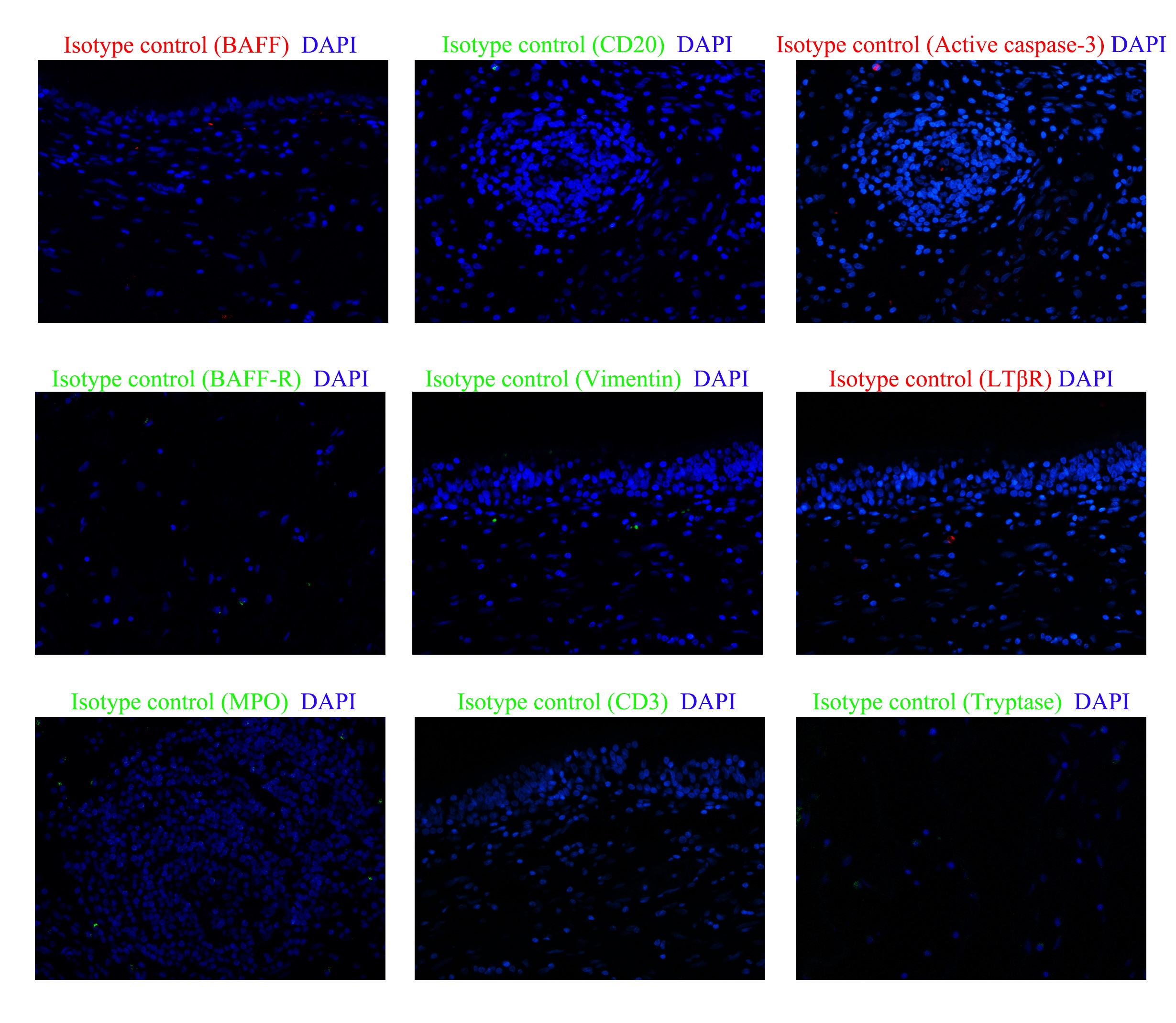

Supplement: Supplementary file 4 [file Image_3.jpeg]
